# Functional phylogenomics analysis of bacteria and archaea using consistent genome annotation with UniFam

**DOI:** 10.1186/s12862-014-0207-y

**Published:** 2014-10-09

**Authors:** Juanjuan Chai, Guruprasad Kora, Tae-Hyuk Ahn, Doug Hyatt, Chongle Pan

**Affiliations:** Computer Science and Mathematics Division, Oak Ridge National Laboratory, Oak Ridge, TN USA; BioSciences Division, Oak Ridge National Laboratory, Oak Ridge, TN USA; Joint Institute for Biological Sciences, University of Tennessee, Knoxville TN, USA

**Keywords:** Prokaryotes, Cellular function, Pathway, Genomes, Evolution, Phylogenomics

## Abstract

**Background:**

Phylogenetic studies have provided detailed knowledge on the evolutionary mechanisms of genes and species in Bacteria and Archaea. However, the evolution of cellular functions, represented by metabolic pathways and biological processes, has not been systematically characterized. Many clades in the prokaryotic tree of life have now been covered by sequenced genomes in GenBank. This enables a large-scale functional phylogenomics study of many computationally inferred cellular functions across all sequenced prokaryotes.

**Results:**

A total of 14,727 GenBank prokaryotic genomes were re-annotated using a new protein family database, UniFam, to obtain consistent functional annotations for accurate comparison. The functional profile of a genome was represented by the biological process Gene Ontology (GO) terms in its annotation. The GO term enrichment analysis differentiated the functional profiles between selected archaeal taxa. 706 prokaryotic metabolic pathways were inferred from these genomes using Pathway Tools and MetaCyc. The consistency between the distribution of metabolic pathways in the genomes and the phylogenetic tree of the genomes was measured using parsimony scores and retention indices. The ancestral functional profiles at the internal nodes of the phylogenetic tree were reconstructed to track the gains and losses of metabolic pathways in evolutionary history.

**Conclusions:**

Our functional phylogenomics analysis shows divergent functional profiles of taxa and clades. Such function-phylogeny correlation stems from a set of clade-specific cellular functions with low parsimony scores. On the other hand, many cellular functions are sparsely dispersed across many clades with high parsimony scores. These different types of cellular functions have distinct evolutionary patterns reconstructed from the prokaryotic tree.

**Electronic supplementary material:**

The online version of this article (doi:10.1186/s12862-014-0207-y) contains supplementary material, which is available to authorized users.

## Background

Bacterial and archaeal microorganisms are capable of very diverse cellular functions. Some examples are nitrogen fixation, organic matter degradation, and antibiotic resistance. The full set of cellular functions, or the functional profile, of a microorganism is encoded in its genome. For a microbial species, some essential cellular functions are maintained via vertical gene transfer, some beneficial ones are gained via horizontal gene transfer or evolutionary innovation, and some dispensable ones are lost in order to maintain a compact genome. Phylogeny and environment are the two primary factors that shape the functional profile of a microbial species. The phylogeny defines the evolutionary history of the species and dictates the cellular functions available from inheritance. The environment, including the physical and chemical conditions of the habitat and the biological cohorts of the microbial communities, applies selective pressure on the species to acquire and retain beneficial cellular functions and remove deleterious or dispensable ones.

Decades of biochemistry research have uncovered the molecular implementation of many cellular functions in terms of biological processes and metabolic pathways. Enzymes that carry out the reactions in pathways and processes have been identified and linked with genes in model organisms in databases, such as MetaCyc, KEGG, and others [1-7]. As a result, many cellular functions in a microorganism can now be inferred computationally from its genome in three steps. First, genes are predicted from the genome sequence. Then, the molecular functions of the genes are inferred by sequence analysis and database searching. Finally, a cellular function is considered to be present in the microorganism if the essential enzymes for the cellular function are encoded in its genome. Owing to the progress in DNA sequencing technology, a large number of genomes (>15,000) have been sequenced and deposited in GenBank [8], covering considerable phylogenetic diversity of the bacterial and archaeal domains. Characterization of cellular functions consistently predicted from the sequenced microbial genomes in a phylogenetic context can shed light on their evolution in Bacteria and Archaea. This is referred to as functional phylogenomics analysis.

Evolution in the bacterial and archaeal domains has been studied extensively based on species trees and gene trees [9,10]. Functional phylogenomics studies can uncover the evolution of cellular functions at an intermediate level between genes and species. Genes may evolve in parallel with species or in a divergent manner through horizontal gene transfers and gene losses. Likewise, a cellular function may evolve in parallel with both its host species and its constituent genes. During this process, some genes may be replaced with their non-homologues of equivalent function, or conferred to a species by horizontal gene transfer, gene sharing, or evolutionary innovation [11-13]. Reconstruction of a cellular function tree is difficult due to the lack of an evolutionary model to account for all these types of events. Instead, the evolution of cellular functions can be studied by inferring their ancestral states across the prokaryotic phylogenetic tree and tracking their gains and losses along the evolutionary timeline. More broadly used cellular functions should have earlier initial appearances in the evolutionary history. We hypothesize that the propagation of a cellular function through the prokaryotic tree may follow the diversification of selected clades, pass laterally across distant species, or discontinue at certain lineages. Our functional phylogenomics analysis provided a quantitative test of these hypotheses for a large number of cellular functions in the phylogenetic tree of all sequenced prokaryotic genomes in GenBank.

Microbiologists have long observed the association of certain functions with certain clades of the phylogenetic tree [14]. Recently, the PICRUSt algorithm [15] was developed to predict metagenome functional profiles of microbial community members from 16S rRNA marker genes. However, many genomics studies have also shown the prevalence of horizontal gene transfer among bacteria [16-20], which would disrupt the association of a cellular function with some closely related taxa. Our functional phylogenomics analysis measured the phylogeny-function correlation for many cellular functions over the prokaryotic tree. On one hand, some functions are concentrated in selected clades. As a result, many clades have distinct functional profiles with enrichment of certain functions. On the other hand, some other functions are sparsely dispersed across distant clades, which would make it difficult to predict the presence or absence of these functions in a microorganism based on the functions of its phylogenetic neighbors. The functional phylogenomics analysis provided the parsimony scores of cellular functions as an empirical measure of their consistency with the phylogeny, and generated the aggregate functional profiles of taxa at different taxonomic ranks.

## Results and discussion

### Phylogeny reconstruction of the prokaryotic genomes

**Figure 1:**
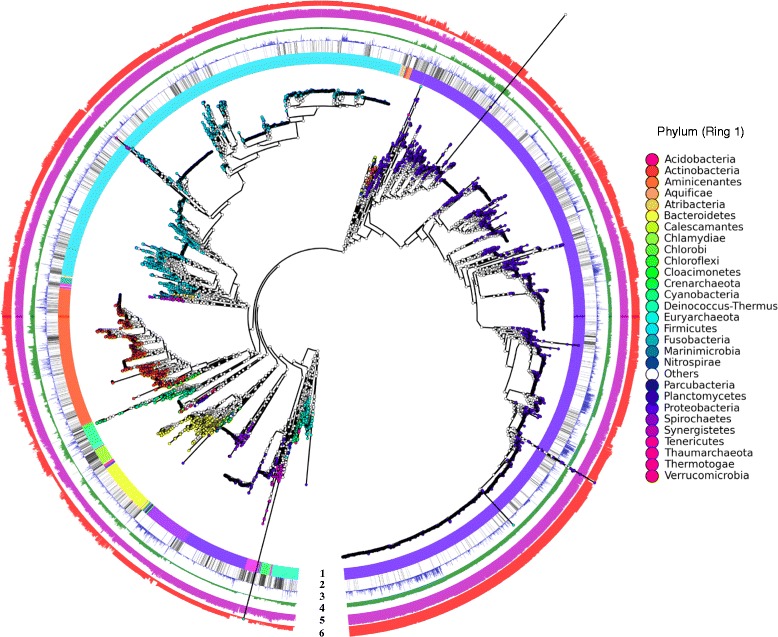
**Overview of the prokaryotic genomes.** The phylogenetic tree contains 14,727 genomes with tips colored according to their phylum classification. Rings: (1) Phylum classification of the genomes. Phyla with less than 5 genomes are in the “Others” category. (2) Completion status of the genomes with black for finished genomes and white for draft genomes. (3) Number of contigs in each genome. (4) Number of proteins in each genome. (5) Percentage of proteins annotated by UniFam in each genome. (6) Number of pathways inferred for each genome.

We downloaded from GenBank (Oct 2013) more than 15,000 bacterial and archaeal genomes, henceforth referred to as the prokaryotic genomes. After discarding genomes containing more than 1000 contigs and genomes with less than 400 protein-coding genes, the remaining 14,727 prokaryotic genomes were analyzed in this study and their metadata was provided in Additional file 1. A phylogenetic tree of these genomes (see Figure 1) was built using PhyloPhlAn [21] and FastTree [22] based on proteins found by Prodigal [23]. Prodigal was chosen in order to standardize gene calling across all genomes, as GenBank gene predictions were shown to have more variation as a result of the different methods used by the submitters [24]. Prodigal predictions were also found to have more consistent identification of translation initiation sites than GenBank records, as in *Burkholderia* genomes [25]. The phylogenetic tree in the Newick format is available in the Additional file 2. The full lineages of the genomes were extracted from the NCBI Taxonomy database and their phyla are color-coded in Ring 1 of Figure 1. Most phyla were monophyletic and occupied contiguous sectors of the Ring 1, except that Proteobacteria was separated into two clades. This indicated a general agreement between the taxonomic classification and phylogenetic distribution of these genomes. Due to the errors in either taxonomic classification or phylogeny reconstruction, there was also occasional inconsistency, indicated by distinct colored lines within some contiguous phylum sections in Ring 1.

To compare the gene calling results from Prodigal predictions and GenBank annotations, we built two phylogenetic trees for the 10,075 genomes that had GenBank annotations. One tree was based on proteins called by Prodigal and the other was based on proteins provided in GenBank (see Additional file 3). The Robinson-Foulds [26] distance between the two trees, defined as the total number of partitions of tips implied by one tree but not the other, was 8494. This was 45% of 18,916 total partitions in the two trees. The branch score [27] defined by the square root of sum of squared differences between branches in the two trees was 2.24, comparing to the total internal branch length of 1074.04 in the two trees. These two distance measures indicated that there were many discordant internal branches between the two trees, but the lengths of these branches were very small.

The taxonomic classification and the phylogenetic tree (see Figure 1) exhibited highly uneven distribution of the sequenced genomes among different phyla. 85% of the genomes belonged to the three most sequenced phyla (7120 genomes in Proteobacteria, 4137 in Firmicutes, and 1294 in Actinobacteria), whereas 17 phyla have 3 or fewer sequenced genomes. The completion status of the genomes is indicated in Ring 2 of Figure 1 (finished genomes in black lines and draft genomes in white lines) and the number of contigs in a genome is shown by the height of its corresponding bar in Ring 3. A few phyla had very high percentages of finished genomes, including Chlamydiae (71% of 146 genomes), Tenericutes (58% of 133 genomes) in the bacterial domain, as did the most phyla in the archaeal domain.

### Re-annotation of the prokaryotic genomes

The prokaryotic genomes were annotated by their submitters to GenBank. We observed a significant degree of inconsistency between the GenBank annotations of different genomes in the following aspects: (i) terminology: genomes have different names for orthologous proteins, (ii) ontology: some genomes have extensive annotations in GO terms and EC numbers and some others do not, and (iii) annotation coverage: closely related genomes have different percentages of genes with assigned functions. The inconsistency probably stemmed from the submitters’ different annotation procedures that may involve a variety of algorithms and databases for inferring functions. In addition, the inconsistency also existed between genomes deposited by the same submitter at different times, because the algorithms and databases used for annotation are continuously improved, but the genome annotation in GenBank was rarely updated after initial submission. Because such inconsistency in the existing GenBank annotations may prevent accurate comparison of cellular functions across genomes, we re-annotated the 14,727 prokaryotic genomes in three steps: gene calling, protein functional annotation, and metabolic pathway inference.

Prodigal [23] was used to predict protein-coding genes in the genomes. The numbers of predicted genes (shown by the height of green bars in Ring 4 of Figure 1) were comparable between neighboring genomes in the phylogenetic tree. The number of genes in a genome was not correlated with the completion status of the genome or its number of contigs. This suggested that, although draft genomes were likely fragmented by many repeat sequences, most of them could still provide sufficient coverage of the gene content of the genomes.

The predicted protein-coding genes were re-annotated using UniFam_Prok. The UniFam protein family database (freely available at http://unifam.omicsbio.org) was constructed from the UniProt database as described in the Material and Methods. The manually curated annotations of proteins in SwissProt were used as the source of function information in standardized terminology. Protein families were built around the SwissProt proteins with their close homologs in TrEMBL. Substantial sequence diversity was obtained for many protein families, owing to the enormous sequence space of TrEMBL. In comparison with many existing protein family databases, UniFam provided the following advantages for genome annotation. First, UniFam provided a comprehensive coverage to alleviate the need for searching multiple databases and combining the results. Ring 5 of Figure 1 shows the percentages of annotated proteins for all the genomes. On average, 70% of the predicted proteins were annotated in a genome. 10,992 out of 14,727 genomes had more than 65% of their proteins annotated by UniFam. Second, because only the manually curated annotations of proteins in SwissProt were used as the source of function information, the UniFam families were associated with standardized protein product names, extensive GO term annotations, and EC numbers. Third, because the UniFam families were built from proteins with stringent whole sequence alignment requirement, we believe the genome annotation by UniFam is reliable. Although it is difficult to rigorously benchmark the accuracy of genome annotation, our manual analysis of selected genomes indicated high quality annotation of UniFam. Finally, the annotation of a microbial genome takes approximately 4 CPU hours on average using UniFam, which made it computationally feasible to re-annotate 14,727 genomes. UniFam will be updated in synchronization with the UniProt database. The annotation of the UniFam families will improve with the ongoing SwissProt curation effort. The sequence diversity of the UniFam families will increase with the expansion of the TrEMBL database.

The UniFam annotation was compared with annotations by RAST [28] and Prokka [29] on five representative genomes: two *Escherichia coli* K-12 genomes (a finished W3110 genome and a draft MG1655 genome), two *Pseudomonas aeruginosa* genomes (a finished M18 genome and a draft SJTD1 genome), and one finished archaeal genome *Methanocaldococcus jannaschii* DSM 2661. The same proteins predicted by Prodigal were provided to the three annotation pipelines. The annotation results in gene names, gene product names and EC numbers from the three pipelines are available in Additional file 4. Only UniFam annotations provided GO terms. UniFam annotated more proteins in the two *E. coli* genomes and the *M. jannaschii* genome, but less in the two *P. aeruginosa* genomes, than Prokka and RAST (see Additional file 5). The consistency between the genome annotations were measured based on EC numbers (see Additional file 6), because two EC numbers can be compared exactly, and the EC number annotation is a primary information source for pathway inference by Pathway Tools. The EC number consistency between the three pipelines was highest in *E. coli*, followed by *P. aeruginosa*, and finally by *M. jannaschii*. The agreement between UniFam and each of the other two methods was better than that between Prokka and RAST. UniFam annotation was more computationally expensive than Prokka and RAST. The *P. aeruginosa* M18 genome was annotated by UniFam using 4.2 CPU hours, by Prokka using 1.3 CPU hours, and by RAST using 0.2 CPU hours.

Metabolic pathways were inferred for all genomes with Pathway Tools [30] based on UniFam annotations. In comparison with pathway inference tools such as KEGG [31] and others [32], Pathway Tools provides a comprehensive coverage of metabolic reactions and pathways [33] and allows automatic processing of a large number of genomes. The pathway inference was facilitated by the extensive EC number and GO term annotation by UniFam. 706 prokaryotic pathways in the MetaCyc database [34] were found in the studied genomes. The numbers of predicted pathways across genomes are shown in Ring 6 of Figure 1. Genomes in the Proteobacteria phylum had more pathways inferred than other phyla, whereas the archaeal genomes had much fewer inferred pathways. The number of inferred pathways in a genome correlated with numbers of predicted and annotated proteins of the genome. In addition, the distribution of curated pathways in MetaCyc among different clades also affected the number of inferred pathways in a genome.

The genome annotation results are provided at http://unifam.omicsbio.org and will be regularly updated to keep pace with the growth of GenBank and newer releases of Prodigal, UniFam and MetaCyc. Although every annotation step was performed with stringent settings to minimize errors, we cannot obtain the ground truth or perform extensive manual curation for such a large set of prokaryotic genomes. Thus, it was not the goal of this study to examine the cellular functions of individual organisms. Instead, the functional phylogenomics analysis was focused on the aggregate results of a large number of diverse genomes to minimize the adverse effect of occasional errors in the phylogenetic tree reconstruction, genome annotation, and pathway inference.

### Association of cellular functions with the taxonomic classification

The overall functional profile of a taxon can be represented by the collection of the biological process GO terms of the proteins encoded in its genomes. The differentiation between the functional profiles of taxa at a given taxonomic rank was measured using GO term enrichment analysis [35]. Because there were too many bacterial genomes for enrichment analysis and result presentation, archaeal genomes were chosen to showcase the differentiation at the phylum and family levels (see Additional file 7). The enrichment analysis was conducted only on taxa containing sufficient genomes: ≥ 10 genomes for a phylum and ≥ 5 for a family. Additional file 7 lists the top ten most enriched GO terms in each phylum compared to the average in the whole archaeal domain, and the top five most enriched GO terms of each family compared to the average in the corresponding phylum.

The Crenarchaeota phylum contains many thermophilic organisms. The enrichment of the biological processes in defense response to viruses indicated that Crenarchaeota has heavier genomic investment in virus defense than the other phyla, which may suggest prevalent viral infection in its environments. Euryarchaeota was specialized in methanogenesis as expected, but it also appeared to have more “intelligence-related” cellular functions, such as signal transduction and transcription regulation. Out of eight families in Euryarchaeota, the five known methanogen families were found to be specialized in methanogenesis along with different sets of other biological processes.

**Figure 2:**
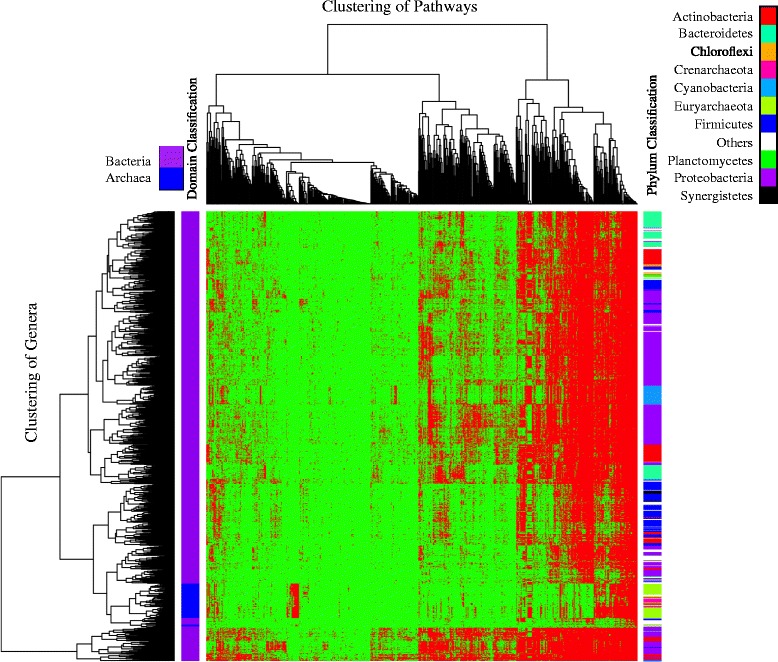
**Hierarchical clustering of genera and metabolic pathways.** The heatmap represents the presence (red) and absence (green) of all 706 pathways (columns) in all 1206 genera (rows). The dendrograms to the left and on the top of the heatmap represent the clustering results of the genera and the pathways, respectively. Higher taxonomic classifications of the genera are marked on the two colored strips: the Bacteria/Archaea domain classification on the left strip and the phylum classification on the right trip.

The metabolic pathway profile of a genome was represented by a 0–1 vector of length 706 with 0 for absence and 1 for presence of the 706 pathways in this genome. The metabolic pathway profiles of individual genomes in a genus were merged into an aggregate genus pathway profile (a pathway profile of a genus pan-genome) to reduce the effect of uneven genera representation on the analysis. The 1206 genera were hierarchically clustered based on their metabolic pathway profiles and 706 metabolic pathways were clustered based on their distribution across the genera (see Figure 2). There were some universal pathways clustered in the right section, which were detected in almost all genera. Examples included tRNA charging, UTP and CTP dephosphorylation I, adenosine ribonucleotides de novo biosynthesis, and glutamine degradation I. Lack of these pathways in the very few genera is likely due to the incompleteness of the genomes. There were also rare pathways that existed only in very few genera, such as quinate degradation II (13 genomes in 2 genera: *Corynebacterium* and *Ketogulonicigenium*) and myrcene degradation (4 genomes in 2 genera: *Gordonia* and *Nocardia*).

The hierarchical clustering of the genera was based on the similarity between their pathway profiles without using the taxonomic or phylogenetic information. The domain and phylum classifications of the genera are marked in the left and right vertical strips, respectively, in Figure 2. The archaeal genera are clearly clustered together. They were distinguished by some Archaea-specific pathways, such as methanogenesis from CO_2_, tRNA splicing, starch degradation V, selenocysteine biosynthesis II, and chitin degradation I. They also lacked some pathways universally found in Bacteria, such as tRNA processing and thiazole biosynthesis I. The clustering of genera was also relatively consistent with their phylum classification, in that the clusters formed continuous blocks of genera from the same phyla. This was a result of many taxa-specific pathways that were commonly found in only a few selected taxa. On the other hand, genera from the same phylum were also often separated into several disjoint clusters, indicating divergence of the functional profiles of genera in the same phylum.

### Association of cellular functions with the phylogenetic tree

**Figure 3:**
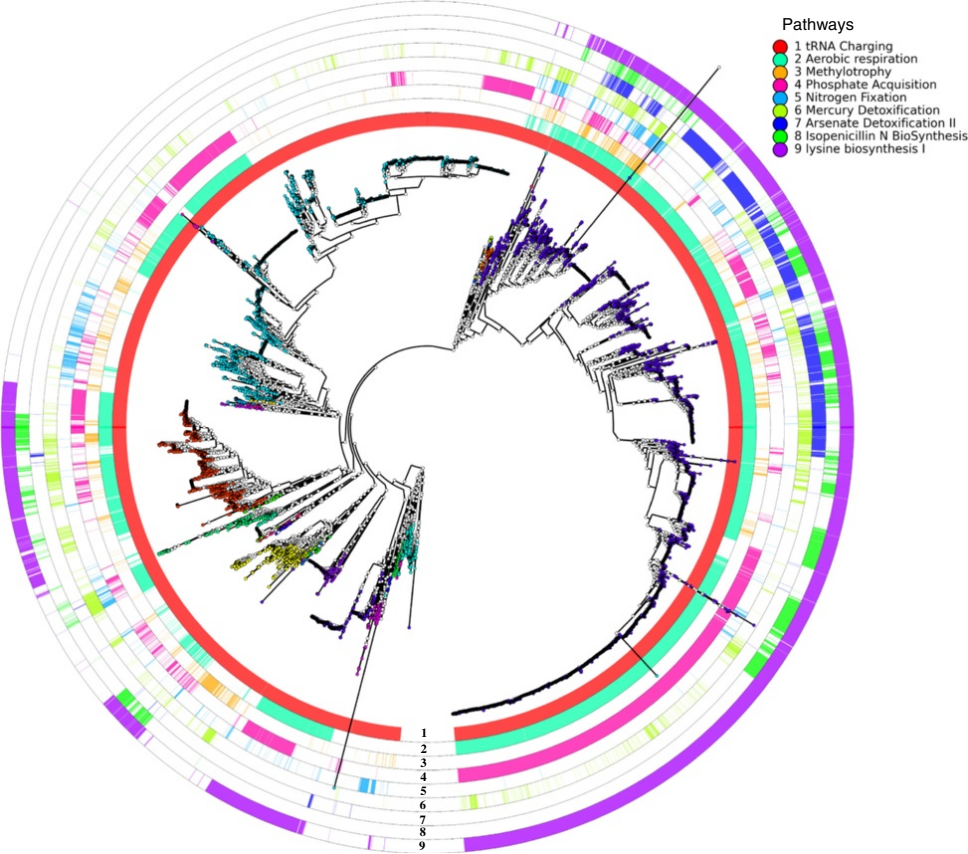
**Distribution of selected pathways across the prokaryotic genomes.** Each ring represents the presence pattern (colored) of a pathway on the phylogenetic tree tips. The pathways and their ring colors are listed in the legend.

The metabolic pathway profiles of genomes were superimposed on their phylogenetic tree to assess the phylogeny-function correlation. Nine representative pathways are shown in Figure 3. The tRNA charging pathway (Ring 1) was found in 99.9% of the genomes and, therefore, was likely to have been passed from an ancient ancestor to all the descendants. The absence of this pathway in 3 genomes can be attributed to the low genome quality, instead of biological effect. The mercury detoxification pathway (Ring 6) existed in only 15.7% of the genomes, but dispersed among many distant clades of the tree. The phylogenetic distribution of this pathway suggested extensive horizontal gene transfer events across the bacterial domain, probably as a result of the selection force of potentially mercury-contaminated environments and the high transferability of this cellular function. In contrast, the arsenate detoxification pathway (Ring 7) found in 14.8% of the genomes was much more concentrated on selected clades. It was not clear why the two heavy-metal detoxification pathways have such different levels of correlation with the phylogeny.

**Figure 4:**
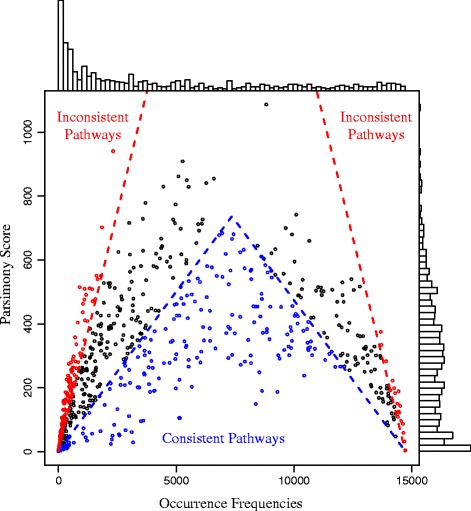
**Distributions of parsimony scores and occurrence frequencies of all pathways.** The pathways are classified into consistent pathways with RI > 0.9 (colored in blue) and inconsistent pathways with RI < 0.7 (colored in red).

A pathway can be viewed as a binary character observed at the tips of the phylogenetic tree, with its presence and absence in a genome as the two states. The parsimony score [36] for a pathway on the tree is the minimum number of state changes (from presence to absence or vice versa) needed along the branches to produce the observed presence/absence pattern of the pathway at the tips. Retention index (RI) [37], which normalizes the parsimony score of a pathway with the number of its occurrences in the genomes, was also calculated to measure how well a character conforms to the phylogenetic tree. A higher RI indicates a better fit of a character to the tree. For example, aerobic respiration (cytochrome C) (Ring 2) was found in 9383 genomes with parsimony score 290 and RI 0.95, whereas methylotrophy (Ring 5) was found in 1353 genomes with a parsimony score 592 and RI 0.56. This is in line with the previous observation that methylotrophy has undergone extensive horizontal gene transfer, while aerobic respiration is consistent with the phylogeny [20]. The parsimony scores and the genome occurrence frequencies of the 706 pathways are shown in Figure 4. Pathways were categorized into consistent pathways with RI > 0.9 and inconsistent pathways with RI < 0.7. Out of the 706 pathways in Bacteria and Archaea, 31% were consistent pathways and 26% were inconsistent pathways. The complete list of the 706 pathways with their parsimony scores, occurrence frequencies in the genomes, and RIs is available in Additional file 8.

Phosphate acquisition was an example of consistent pathways (Ring 4). This pathway was found in 6005 genomes with parsimony score 528 and RI 0.91. It was concentrated in 16 bacterial phyla and 2 archaeal phyla. Of the bacterial genomes, it was mostly found in Proteobacteria (3882 out of 7120 genomes), Firmicutes (1343 out of 4137), Actinobacteria (434 out of 1294), Bacteroidetes (170 out of 488), and Cyanobacteria (69 out of 173). Nitrogen fixation was an example of inconsistent pathways (Ring 5). It was found in 1121 genomes with parsimony score 409 and RI 0.64. This pathway is known to have been horizontally transferred between many species [20]. Although both phosphate acquisition and nitrogen fixation provide essential nutrients for microorganisms, they have very different occurrence frequencies and horizontal gene transfer behaviors in the bacterial genomes.

Two biosynthesis pathways are also shown in Figure 3. Isopenicillin N biosynthesis (Ring 8) occurred in 2236 genomes with parsimony score 387 and RI 0.83. Lysine biosynthesis I (Ring 9), which involves succinylated intermediates, was found in 8372 genomes with parsimony score 149 and RI 0.97, showing much higher prevalence and consistency. The two pathways were both mostly found in the phyla of Proteobacteria, Actinobacteria and Bacteroidetes. However, isopenicillin N biosynthesis had a very sparse distribution, while lysine biosynthesis I had a nearly uniform distribution in these phyla.

From the presence/absence pattern of a pathway at the tips of the phylogenetic tree, the ancestral states of the pathway at the internal nodes of the tree were inferred with the parsimony method [36,38,39]. The divergence time of the internal nodes of the tree was also estimated [40] with a scaled reference age of 100 for the root. Combining the ancestral states of a pathway and the divergence time at internal nodes, the state changes (gain or loss) of the pathway were clocked on the phylogenetic tree.

**Figure 5:**
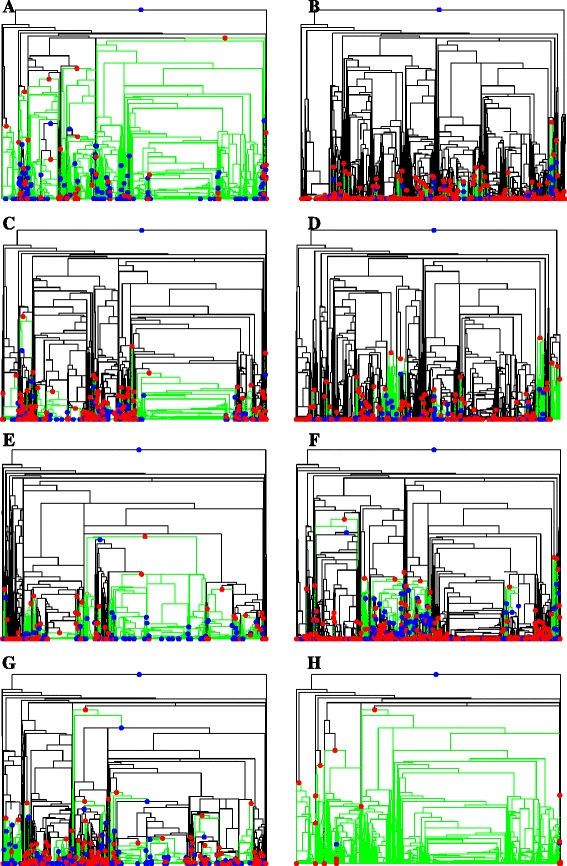
**Subtrees of selected pathways.** The subtree of a pathway is reduced from the clocked phylogenetic tree of all genomes by collapsing the entire clades without this pathway into tips. The root is colored red for the pathway’s presence and blue for its absence. The colors of non-root nodes mark the pathway’s status changes from their immediate ancestral nodes: red for gains, blue for losses, and none for no change. The branches descending from nodes containing the pathway are colored green. The total number of blue and red nodes in a pathway’s subtree equals the parsimony score of the pathway. **(A)** Aerobic respiration (cytochrome C) with parsimony score 290 from 9383 genomes. **(B)** Methylotrophy with parsimony score 592 from 1353 genomes. **(C)** Phosphate acquisition with parsimony score 528 from 6005 genomes. **(D)** Nitrogen fixation with parsimony score 409 from 1121 genomes. **(E)** Arsenate detoxification with parsimony score 196 from 2182 genomes. **(F)** Mercury detoxification with parsimony score 944 from 2319 genomes. **(G)** Isopenicillin N biosynthesis with parsimony score 387 from 2236 genomes. **(H)** Lysine biosynthesis I with parsimony score 149 from 8372 genomes.

Figure 5 shows four pairs of subtrees for the representative pathways examined in Figure 3. Without considering horizontal gene transfer from eukaryotes, the first red node from the root on the subtree for a pathway marks the first occurrence of the pathway in nature through evolutionary innovation. The subsequent red nodes may result from independent evolutionary innovation or horizontal gene transfer. The first pair (5A and B) displayed very distinct evolutionary patterns. Aerobic respiration had very early first appearance and was well maintained in most clades, whereas methylotrophy was a more recent innovation and underwent many changes. The second pair (Figure 5C and D) had similar parsimony scores (528 and 409), but very different genome occurrence frequencies (6005 and 1121). The gains of phosphate acquisition were concentrated in only a few clades, while the gains of nitrogen fixation were spread across many clades. The third pair (Figure 5E and F) shows two heavy-metal detoxification pathways. The arsenate one showed a small number of losses and gains in a few clades, while the mercury one had a few ancient gains and losses followed by considerable recent gains and losses in many clades. For the two biosynthesis pathways (Figure 5G and H), isopenicillin N biosynthesis went through frequent gains and losses, probably in response to selection pressure in specific environments; whereas lysine biosynthesis I was maintained stably with few losses in almost all clades since their ancestors first gained this pathway.

## Conclusions

The phylogenetic tree and consistent annotations of 14,727 prokaryotic genomes provided the foundation for a functional phylogenomics analysis across Bacteria and Archaea. To our best knowledge, this is the first study that systematically examined a wide variety of consistently inferred cellular functions across prokaryotic genomes in a phylogenetic context. The analysis systematically measured the correlation of the cellular function evolution with the organism evolution in terms of function enrichment in taxa, clustering of functional profiles and genera, and parsimony score distribution of metabolic pathways. Different evolution patterns of cellular function were showcased using the phylogenetic distributions and subtrees of selected metabolic pathways.

## Methods

### Construction of the UniFam database

All proteins between 30 and 5000 residues long were collected from UniProt (535,849 proteins from SwissProt and ~42 million proteins from TrEMBL, accessed in October 2013). The 43 million proteins were sorted by length in decreasing order and then clustered into ~13 million clusters using 64-bit USEARCH v7.0.1001 [41] on a Linux workstation with 130GB of memory. The clustering required every sequence in a cluster to have >80% global alignment identity with the centroid sequence of the cluster, which guaranteed >60% global alignment identity between any two sequences in a cluster. And the shortest sequence was required to be >80% as long as the longest sequence in a cluster. These two requirements were used to prevent clustering of proteins that were only locally similar. Default values for other parameters of USEARCH were used for clustering.

267,579 clusters containing at least one SwissProt protein were used to construct UniFam families. The UniFam clusters included all SwissProt proteins and ~23% of all proteins from UniProt. Additional file 9A shows the distribution of the numbers of UniProt proteins in the UniFam clusters. The average size of the UniFam clusters was 37 proteins and the biggest cluster contained 27,467 proteins. Singletons constituted 27% of the clusters, but covered only 0.7% of all proteins included in UniFam. 33,940 singletons were from Bacteria, 28,953 from Eukaryota, 6138 from Archaea, and 2026 from Viruses. The organelle distribution of the singletons was 1703 in plastids, 652 in plasmids, 466 in mitochondria, 7 in nucleomorphs, 4 in hydrogenosomes, and the remaining in chromosomes.

For non-singleton clusters, MAFFT v7.113b [42] was used to create their multiple sequence alignments (MSAs). MAFFT was set to automatically select an appropriate strategy according to the cluster size. Because of the high pairwise global alignment identities between sequences in a cluster, even large MSAs were observed to have low alignment uncertainty. HMMs were built using HMMER 3.1b1 [43] either from single sequences for singleton clusters, or from MSAs for non-singleton clusters, with default options. The distribution of the HMM lengths was centered at 250 residues with a long tail for long HMMs (see Additional file 9B). These two steps were executed on the supercomputer, Titan, at the Oak Ridge Leadership Computing Facility.

The following fields from the annotations of the SwissProt proteins in a cluster were extracted and used for the annotations of the corresponding UniFam family: recommended full name, EC number, gene name, and GO terms. Database-specific annotations, such as Pfam IDs [44] and COG categories [45], were not used, because they should be best assigned by searching those specific databases. If there was more than one SwissProt protein in a cluster and their annotations were not the same for an annotation field, the union was taken for that field as the annotation for the cluster. We observed very consistent annotations between SwissProt proteins in a cluster, because of the high sequence identity between proteins in a cluster and the reliability of SwissProt annotation.

Two sub-databases were created: UniFam_Prok (159,895 families) for annotating prokaryotic proteins and UniFam_Euk (107,777 families) for annotating eukaryotic proteins. The smaller sub-databases allowed faster database searching. Families with bacterial and archaeal proteins were combined to form UniFam_Prok and those with eukaryotic proteins formed UniFam_Euk. The few families that had proteins from both prokaryotes and eukaryotes were included in both sub-databases. However, only the annotations for proteins from prokaryotic organisms were transferred to UniFam_Prok and likewise for UniFam_Euk. To illustrate the functional coverage of UniFam, the distributions of the EC number assignment to UniFam families were plotted for UniFam_Euk (see Additional file 10A) and UniFam_Prok (see Additional file 10B).

### Re-annotation and pathway reconstruction of prokaryotic genomes

All bacterial and archaeal genomes, in finished or draft completion status, were downloaded from GenBank [8] (Oct 2013). Highly fragmented genomes with more than 1000 contigs were discarded. Every genome was annotated in the following steps. First, Prodigal [23] was used to find protein-coding genes and predict their protein sequences. Genomes with less than 400 predicted genes were also discarded. The proteins were then searched against UniFam_Prok using HMMER 3.1b1 [43]. The top matches (ranked by whole-sequence e-value) of proteins were selected by requiring the whole-sequence e-value to be lower than 1.00E-3 and the aligned regions (not necessarily contiguous) to cover at least 50% of both the matched HMM and the query protein. Proteins were annotated with the function information of their best-matched UniFam families.

For the comparison between Prokka, RAST and UniFam in the five representative genomes, Prokka was run without rRNA and tRNA searching using eight threads (2.8-GHz AMD Opteron processors), Macintosh myRAST was run with default settings (3.4-GHz quad-core Intel i7 processor), and UniFam was run using two threads (2.3-GHz AMD Opteron processors). Proteins predicted by Prodigal were provided to all three pipelines as input.

The UniFam annotations of genomes in GenBank-format were used to reconstruct metabolic pathways with the PathoLogic module in Pathway Tools v17.5 [30]. PathoLogic was run in the batch mode, without hole filler, cellular overview graph, or patch download. MetaCyc [34] was used as the reference database and only pathways whose predicted taxonomic range includes prokaryotes were considered. From the output pathways report, only the pathways with confidence factors ≥70 were kept for the functional phylogenomics analysis.

### Functional phylogenomics analysis

The R package, topGO [35,46], was used to find the biological process GO terms that were enriched at two taxonomic ranks for Archaea: phyla with more than 10 genomes and families with more than 5 genomes. The enrichment in phyla and families were relative to the Archaea domain and the corresponding phyla, respectively. Only GO terms annotated to more than 10 genes were included. “Classic”, “weight”, and “elim” algorithms with Fisher’s statistic were performed. GO terms with >0.01 *p*-values calculated by the “elim” algorithm were discarded to eliminate very general and low-level GO terms. The most enriched GO terms shown in Additional file 7 were ranked by the *p*-values calculated by the “weight” algorithm.

Hierarchical clustering was performed on a 0–1 matrix with the dimension of 706 (pathways) by 1206 (genera). A pathway was considered to be present in a genus with a value of 1 in the matrix, if this pathway was inferred for any genome in this genus. Manhattan distance was used for the distance function in clustering, which was defined as the total number of different entries between 2 vectors. The clustering and plotting were performed with the function “heatmap.2” in the R package gplots [47].

PhyloPhlAn [21] was used to reconstruct the phylogenetic tree of all prokaryotic genomes based on the gene prediction results from Prodigal. After the alignments of 400 conserved genes were generated and concatenated into a long protein sequence alignment, FastTree v2.1.7 [22] was run separately to build the phylogenetic tree with Normal + NNI + SPR search and Jones-Taylor-Thorton [48] maximum likelihood model for amino acid evolution. The sequence alignment and the tree building were completed in 10.4 hours and 7.1 hours, respectively, using 64 threads on a Linux machine with four 2.3-GHz 16-core AMD Opteron processors. The circular tree figures were produced using GraPhlAn (https://bitbucket.org/nsegata/graphlan/wiki/Home).

The Robinson-Foulds [26] distance and branch scores [27] between the two phylogenetic trees for the 10,075 genomes were calculated with the R package APE [49].

Each pathway was mapped to the phylogenetic tree as a binary character and its parsimony score was calculated with the R package phangorn [38]. Divergence times in the phylogenetic tree of genomes were estimated using PATHd8 [40], with scaled reference age at the root set to 100. Retention Index (RI) [37] of a character on a phylogenetic tree was defined as (g-s)/(g-m), where g = maximum number of changes possible for a character, s = number of changes observed on the tree, m = minimum number of changes possible for a character. In our calculation of the RI of a pathway, g was the number of genomes with this pathway or the number of genomes without this pathway, whichever was smaller; s was the parsimony score of this pathway; and m was 1 if both presence and absence states were observed in the genomes, and 0 if this pathway was present in all genomes.

### Availability of supporting data

The data sets supporting the results of this article are included within the article and its additional files.

## Additional files

Additional file 1
**Metadata for the studied prokaryotic genomes.**
Additional file 2
**Phylogenetic tree of prokaryotic genomes.** Newick format files can be opened by standard tree viewing programs, and most phylogenetic programs. Additional file 3
**Comparison of phylogenetic trees reconstructed from two different sets of proteins.** **(A)** Tree based on existing genes in GenBank. **(B)** Tree based on proteins predicted by Prodigal. Both trees contain 10,075 genomes. The tips and the rings are colored according to phylum classification of the genomes. Phyla with less than 5 genomes are grouped in the “Others” category. Additional file 4
**Functional annotations by Prokka, RAST and UniFam in representative genomes.**
Additional file 5
**Comparison of functional annotations by Prokka, RAST and UniFam in representative genomes.**
Additional file 6
**Consistency of EC number assignments between Prokka, RAST and UniFam in representative genomes.**
Additional file 7
**Top ten enriched GO terms for the archaeal phyla and top five enriched GO terms for the archaeal families.**
Additional file 8
**Parsimony scores, occurrence frequencies and retention indices of metabolic pathways.**
Additional file 9
**Summary statistics of the UniFam database.** **(A)** Histogram of sizes of UniFam families. **(B)** Histogram of the HMM lengths of UniFam families. Additional file 10
**Bubble plots of EC numbers in the UniFam database and the studied prokaryotic genomes.** Each bubble represents the first three fields of an EC number. The first field is represented by the colors of the bubbles, and the second field and third field by the x- and y- coordinates of the bubbles, respectively. The dash sign in a field is assigned with a value of 0. The areas of the bubbles are proportional to the frequencies of the corresponding EC numbers in each plot. (A) UniFam_Euk sub-database. EC 2.7.11.- has the highest frequency of 2220. (B) UniFam_Prok sub-database. EC 2.1.1.- has the highest frequency of 5958. (C) Annotated proteins in the prokaryotic genomes. EC 2.7.1.- has the highest frequency of 839,278.

## Abbreviations

BP: Biological process
CDS: Coding sequences/regions
EC: Enzyme classification
GO: Gene ontology
HMM: Hidden markov model
MSA: Multiple sequence alignment
RI: Retention index
