# Supplementary material for: Functional phylogenomics analysis of bacteria and archaea using consistent genome annotation with UniFam
Source: BMC Evol Biol. 2014 Oct 9;14:207. doi: 10.1186/s12862-014-0207-y (PMC4194380; doi:10.1186/s12862-014-0207-y)
Supplement: Additional file 3 — Comparison of phylogenetic trees reconstructed from two different sets of proteins. (A) Tree based on existing genes in GenBank. (B) Tree based on proteins predicted by Prodigal. Both trees contain 10,075 genomes. The tips and the rings are colored according to phylum classification of the genomes. Phyla with less than 5 genomes are grouped in the “Others” category. [file 12862_2014_207_MOESM3_ESM.pdf]

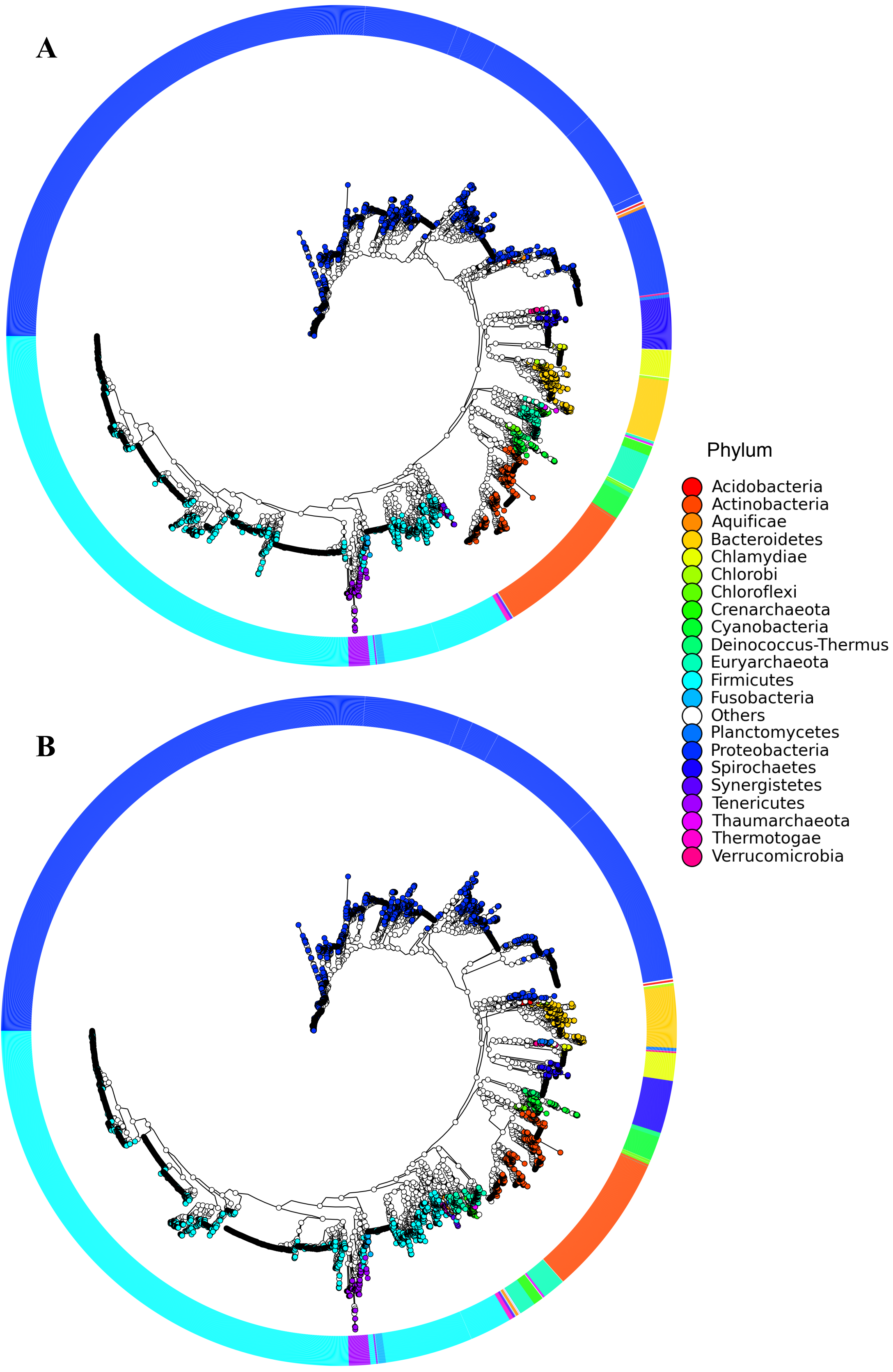
A
B
Phylum
Acidobacteria
Actinobacteria
Aquificae
Bacteroidetes
Chlamydiae
Chlorobi
Chloroflexi
Crenarchaeota
Cyanobacteria
Deinococcus-Thermus
Euryarchaeota
Firmicutes
Fusobacteria
Others
Planctomycetes
Proteobacteria
Spirochaetes
Synergistetes
Tenericutes
Thaumarchaeota
Thermotogae
Verrucomicrobia
