# Supplementary figures and images for: Functional phylogenomics analysis of bacteria and archaea using consistent genome annotation with UniFam
Source: BMC Evol Biol. 2014 Oct 9;14:207. doi: 10.1186/s12862-014-0207-y (PMC4194380; doi:10.1186/s12862-014-0207-y)

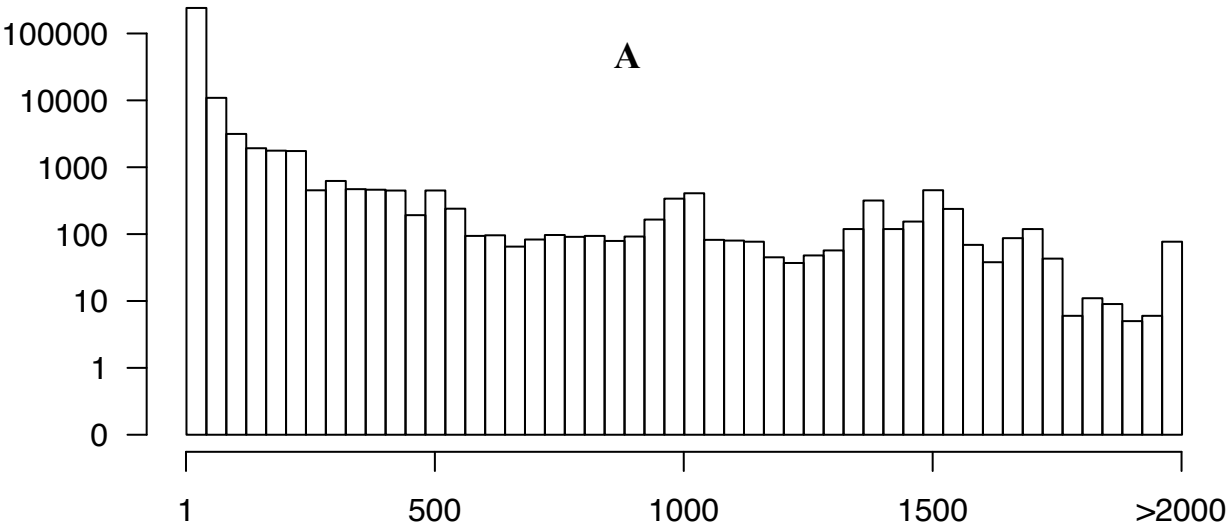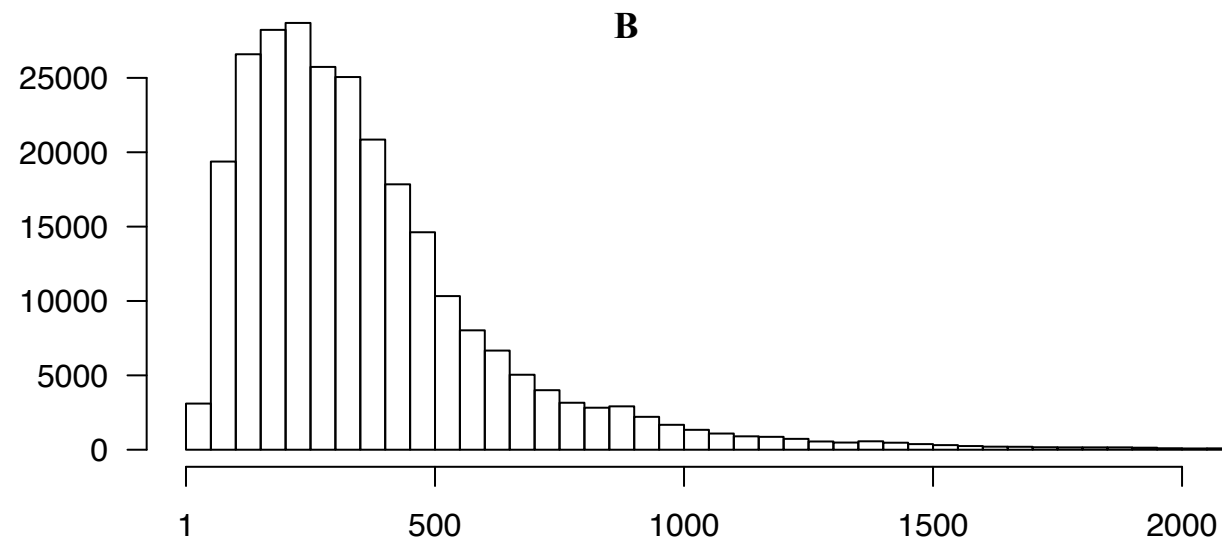

Supplement: Additional file 9 — Summary statistics of the UniFam database. (A) Histogram of sizes of UniFam families. (B) Histogram of the HMM lengths of UniFam families. [file 12862_2014_207_MOESM9_ESM.pdf]

**A**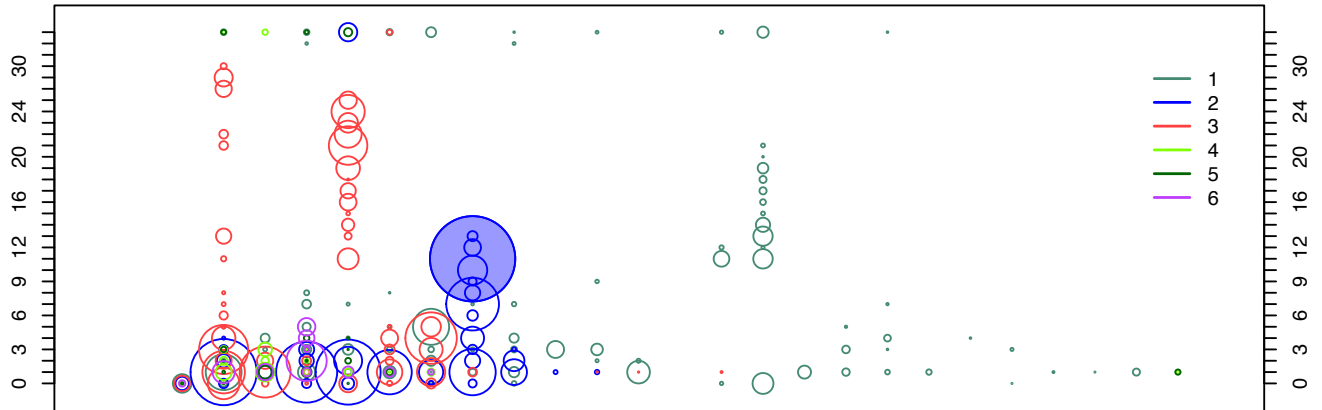**B**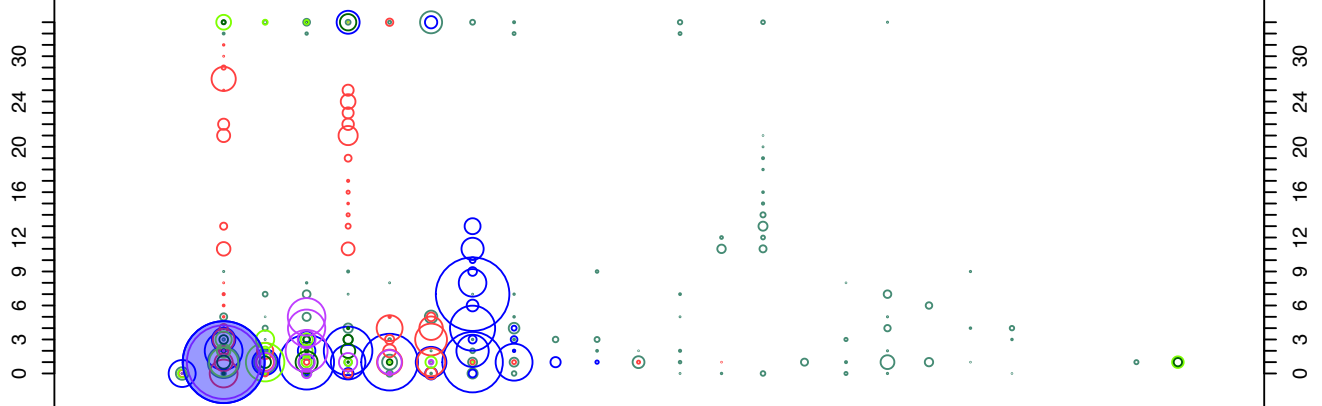**C**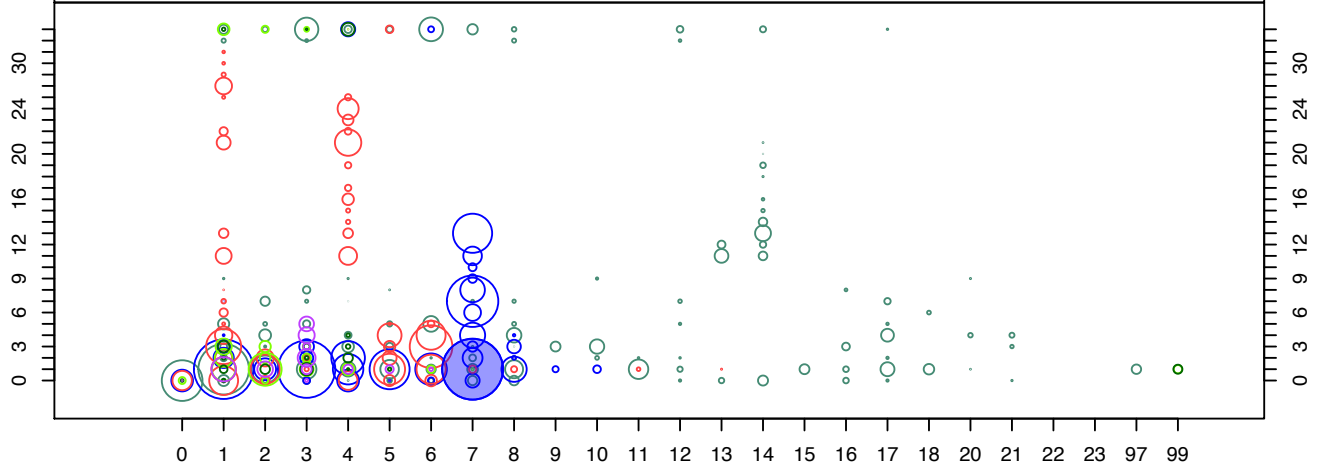

Supplement: Additional file 10 — Bubble plots of EC numbers in the UniFam database and the studied prokaryotic genomes. Each bubble represents the first three fields of an EC number. The first field is represented by the colors of the bubbles, and the second field and third field by the x- and y- coordinates of the bubbles, respectively. The dash sign in a field is assigned with a value of 0. The areas of the bubbles are proportional to the frequencies of the corresponding EC numbers in each plot. (A) UniFam_Euk sub-database. EC 2.7.11.- has the highest frequency of 2220. (B) UniFam_Prok sub-database. EC 2.1.1.- has the highest frequency of 5958. (C) Annotated proteins in the prokaryotic genomes. EC 2.7.1.- has the highest frequency of 839,278. [file 12862_2014_207_MOESM10_ESM.pdf]
